# Comparative analysis of the anticoagulant activities and immunogenicity of HSC70 and HSC70^TKD^ of *Haemaphysalis flava*

**DOI:** 10.1186/s13071-022-05521-2

**Published:** 2022-11-05

**Authors:** Yu-Ke Liu, Guo-Hua Liu, Lei Liu, Ai-Bing Wang, Tian-Yin Cheng, De-Yong Duan

**Affiliations:** Research Center for Parasites & Vectors, College of Veterinary Medicine, Hunan Agricultural University, Changsha, 410128 Hunan Province China

**Keywords:** *Haemaphysalis flava*, HSC70, Site-directed mutagenesis, Anticoagulation, Immunogenicity

## Abstract

**Background:**

*Haemaphysalis flava* is a hematophagous ectoparasite that acquires the nutrition needed for development and reproduction by sucking blood and digesting the blood meal. During blood-sucking and blood-meal digestion, the prevention of blood coagulation is important for this tick. Previous studies have shown that heat shock cognate 70 (HSC70) protein has certain anticoagulant activities, but its immunogenicity remains unclear. Also, whether the mutation of individual bases of the TKD-like peptide of HSC70 through the overlap extension method can change its anticoagulant activities and immunogenicity remains to be investigated.

**Methods:**

The gene encoding the HSC70 protein was cloned from a complementary DNA library synthesized from *H. flava*. The coding gene of the TKD-like peptide of HSC70 was mutated into a TKD peptide coding gene (HSC70^TKD^) using the overlap extension method. *Escherichia coli* prokaryotic expression plasmids were constructed to obtain the recombinant proteins of HSC70 (rHSC70) and HSC70^TKD^ (rHSC70^TKD^). The purified rHSC70 and rHSC70^TKD^ were evaluated at different concentrations for anticoagulant activities using four in vitro clotting assays. Emulsifying recombinant proteins with complete and incomplete Freund’s adjuvants were subcutaneously immunized in Sprague Dawley rats. The serum antibody titers and serum concentrations of interferon-gamma (IFN-γ) and interleukin-4 (IL-4) were detected using an indirect enzyme-linked immunosorbent assay to assess the immunogenicity of rHSC70 and rHSC70^TKD^.

**Results:**

The open reading frame of HSC70 was successfully amplified and found to have a length of 1958 bp. The gene encoding the TKD-like peptide of HSC70 was artificially mutated, with the 1373-position adenine (A) of the original sequence mutated into guanine (G), the 1385-position cytosine (C) mutated into G and the 1386-position G mutated into C. rHSC70 and rHSC70^TKD^ that fused with His-tag were obtained using the expression plasmids pET-28a-HSC70 and pET-28a-HSC70^TKD^, respectively. rHSC70 and rHSC70^TKD^ prolonged the thrombin time (TT) and reduced the fibrinogen (FIB) content in the plasma, but did not affect the prothrombin time (PT) or activated partial thromboplastin time (APTT) when compared to the negative control. Interestingly, the ability of rHSC70^TKD^ to prolong the TT and reduce the FIB content in the plasma was better than that of rHSC70. The specific antibody titers of both rHSC70 and rHSC70^TKD^ in rat serum reached 1:124,000 14 days after the third immunization. The serum concentration of IFN-γ in the rHSC70^TKD^ group was higher than that in the rHSC70 group. The rHSC70 group has the highest serum concentration of IL-4, and the serum concentration of IL-4 in the rHSC70^TKD^ group was higher than that in the negative group.

**Conclusions:**

rHSC70 and rHSC70^TKD^ exhibited anticoagulant activities by prolonging the TT and reducing the FIB content in vitro. rHSC70^TKD^ had better anticoagulant activities than rHSC70. Both rHSC70 and rHSC70^TKD^ had good immunogenicity and induced humoral and cellular immunity.

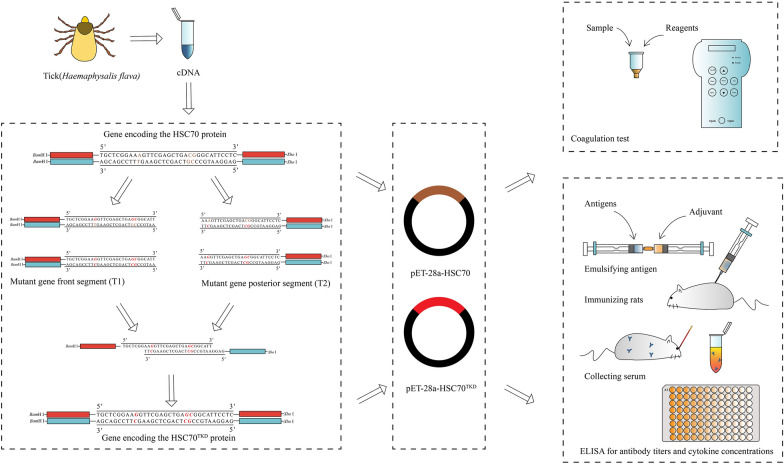

## Background

*Haemaphysalis flava* ticks are widely distributed throughout southern China and many countries in Southeast Asia [1–3]. This tick species feeds on humans, cattle, boar, pandas and other mammals as common hematophagous ectoparasites and can carry bacteria, including *Rickettsia*, *Anaplasma*, *Coxiella burnetti*, *Ehrlichia*, *Francisella tularensis*, *Borrelia theileri* or *Bartonella* [4–7], and viruses, including severe fever with thrombocytopenia syndrome virus [8], tick-borne phleboviruses, Kabuto Mountain viruses [9] and Tarumizu tick viruses [10]. These pathogens are threats to human health and animal husbandry. Traditional control methods against ticks are based on the use of chemical insecticides, but the effects of drug residues in the diet and pollution of the environment cannot be underestimated [11, 12]. Therefore, sifting through premium potential antigens to develop a broad-spectrum vaccine against ticks is urgently needed.

Heat shock proteins (HSPs) are evolutionarily conserved chaperones that are present extensively in prokaryotes and eukaryotes [13]. HSPs play roles in the folding, assembly, degradation and repair of intracellular proteins, in antigen-presenting cells and in the recognition and regulation of immune responses [14]. Previous studies found that rats administered Chinese medicine that promotes blood circulation exhibited significantly upregulated expression levels of HSP70 messenger RNA (mRNA) in the serum [15]. It has also been reported that it is easier to introduce blood stasis in HSP70 knockout mice [16] and that as ticks become rapidly engorged, the mRNA expression of HSP70-8 (heat shock cognate 70 [HSC70]) in the midgut and salivary gland is significantly upregulated [17]. These findings suggest that some HSP70 family members possess anticoagulation activities. Therefore, we hypothesized that the tick-born HSC70 protein is a potential antigen that stimulates the animal body to produce corresponding antibodies, which in turn disable the effects of tick-born HSC70, thereby affecting tick blood-sucking and blood-meal digestion and ultimately achieving control of ticks.

Exogenous HSP70 stimulates T cells and B cells to initiate immune system activities by generating peptides to major histocompatibility complex class I and class II (MHC I and II) molecules for antigen presentation [18–22]. However, in a previous study, the immunization of rabbits with the recombinant HSP70 protein from *Haemaphysalis longicornis* did not protect the rabbits against ticks [23]. This result may be due to the variation in the TKD peptides of HSP70 family members in ticks, which are TKD-like peptides [24]. A previous study confirmed that HSC70 has TKD-like peptides that fail to activate NK cell activity, including the 14-mer TKDNNLLGKFELTG, while HSP70 has TKD peptides, including the 14-mer TKDNNLLGRFELSG, which do stimulate the proliferative activity of NK cells [25]. In the present study, we constructed a plasmid to express recombinant protein HSC70 (rHSC70) using an *Escherichia coli* prokaryotic expression system. Through the overlap extension method, we acquired an HSC70 gene of *H. flava* with mutated TKD peptide sites, which also ligated to an *E*. *coli* expression plasmid to obtain the recombinant protein of HSC70^TKD^, rHSC70^TKD^. After purifying rHSC70 and rHSC70^TKD^, we evaluated the anticoagulation activities and immunogenicity.

## Methods

### Total RNA extraction

All *H. flava* samples were collected from the body surface of hedgehogs in the city of Xinyang in Henan Province (31°53′N, 114°04′E), China, and identified using a stereomicroscope based on previously described morphological characteristics [26]. The surfaces of the ticks were cleaned and sterilized with 75% ethanol. Total RNA was extracted using an EasyPure RNA Kit (TransGen Biotech, Beijing, China). The quality of RNA was assessed by electrophoresis in a 1% agarose gel. RNA samples with clear and distinct electrophoretic bands as a template were selected for synthesizing *H. flava* complementary DNA (cDNA) using TransScript® II All-in-One First-Strand cDNA Synthesis SuperMix for PCR (TransGen Biotech).

### Cloning of the open reading frame of the HSC70 gene and site-directed mutation

Based on the HSC70 gene sequences of *H. flava* (GenBank accession No. KM111606), primers amplifying the HSC70 and mutant HSC70^TKD^ genes were designed using Primer Premier v5.0 and synthesized by Sangon Biotech (Shanghai, China) (Table 1). The primers HSC70-f-*Bam*H and HSC70-r-*Xho*I were used to amplify the open reading frame (ORF) sequences of HSC70. The PCR assays were carried out in a reaction volume containing 2 μl each of forward and reverse primers (10 mM), 1 μl cDNA template, 25 μl Premix Taq (Takara, Dalian, China) and 20 μl ddH_2_O. The amplification conditions consisted of an initial denaturation at 95 °C for 5 min, followed by 31 cycles at 95 °C for 30 s, 60 °C for 40 s and 72 °C for 1 min, with a final extension at 72 °C for 7 min. The PCR products were purified using an Agarose Gel DNA Extraction Kit (TransGen Biotech) following the manufacturer’s instructions for DNA cloning and sequencing.

**Table 1:** Primer sequences

Primer names	Primer sequences^a^
HSC70-f-*Bam*H	5′—cgGGATCCGCGAAGGTGCCCGCAATT -3′
HSC70-r-*Xho*I	5′—ccgCTCGAGATCCACTTCTTCAATTGTGGGCCCACT -3′
TKD-r	5′—AA**G**GTTCGAGCTGA**GC**GGCATTCCTC -3′
TKD-f	5′—AATGCC**GC**TCAGCTCGAAC**C**TTCCGAGCA -3

^a^The lowercase letters of primer HSC70-f-*Bam*H and HSC70-r-*Xho*I are protected bases. The underlined sequences of primer HSC70-f-*Bam*H and HSC70-r-*Xho*I are cleavage sites of restriction enzymes* Bam*H and* Xho*I. The letters in primer TKD-r and TKD-f in bold font represent mutation sites

The PCR products of the ORF of HSC70 were diluted as a template for amplifying two mutant DNA fragments consisting of HSC70^TKD^. The primers HSC70-f-*Bam*H and TKD-r were used to amplify the front segment of the mutated gene (T1). The reaction volume was the same as described above. The amplification conditions consisted of an initial denaturation at 95 °C for 5 min, followed by 30 cycles at 95 °C for 30 s, 58 °C for 40 s, 72 °C for 30 s, with a final extension at 72 °C for 7 min. The primers TKD-f and HSC70-r-*Xho*I were used to amplify the posterior segment of the mutant gene (T2). The reaction volume and amplification conditions were the same as for T1. The T1 and T2 fragments were ligated using In-Fusion HD Cloning Plus (Takara) according to the manufacturer’s instructions. With the reaction products as the template, the primers HSC70-f-*Bam*H and HSC70-r-*Xho*I were used to amplify the ORF of HSC70^TKD^. The reaction volume contained 2 μl each of the forward and reverse primers (10 mM), 2 μl of template, 25 μl Premix Taq (Takara) and 19 μl ddH_2_O. The amplification conditions were the same as those for the ORF of HSC70. The products of HSC70^TKD^ ORF were purified as described above. The PCR products and ORFs of HSC70 and HSC70^TKD^ were cloned using a pMD™ 19-T Vector Cloning Kit (Takara) according to the manufacturer’s instructions. The correctly identified cloned plasmids were named pMD19-T-HSC70 and pMD19-T-HSC70^TKD^, which were extracted using a TIANprep Mini Plasmid Kit (TransGen Biotech) and sequenced by Sangon Biotech.

### Construction of the HSC70 and HSC70^TKD^ expression plasmids

Following the digestion of the pMD19-T-HSC70 and pMD19-T-HSC70^TKD^ plasmids with the *Bam*HI and *Xho*I restriction enzymes (Takara), the products were ligated to linearized pET-28a vectors with the same restriction enzyme digestion using a DNA Ligation Kit (Accurate Biotech, Hunan, China). The ligation solutions were transformed into *Trans* BL21 (DE3) competent cells (TransGen Biotech). The correctly identified cloned plasmids were named pET-28a-HSC70 and pET-28a-HSC70^TKD^, following selection on LB plates containing kanamycin (100 μg/ml), verified using the *Bam*HI and *Xho*I restriction enzymes and sequenced by Sangon Biotech.

The selected single colonies containing the pET-28a-HSC70 or pET-28a-HSC70^TKD^ plasmids were inoculated in LB media containing kanamycin (50 μg/ml), then cultured in an incubator shaker (Thermo Fisher Scientific, Waltham, MA, USA) at 37 °C at 1.1 *g* until 0.6 OD_600nm_ was reached. Isopropyl β-d-1-thiogalactopyranoside (IPTG) was added to induce expression, and a bacterial solution without IPTG in each group was reserved as control groups. Then, a 1-ml sample was collected from the IPTG and control groups, respectively, after 2, 4 and 6 h. All samples were centrifuged at 8608.6 *g* for 1 min to enrich the bacteria, then 50 μl ddH_2_O was added for resuspension. All samples were detected by sodium dodecyl sulfate–polyacrylamide gel electrophoresis (SDS-PAGE) to determine the time of maximum protein expression.

The bacteria expressing rHSC70 or rHSC70^TKD^ were cultivated and collected under the same conditions as described above. The collected bacteria were resuspended in lysis buffer (20 mM Tris, pH 7.4, 250 mM NaCl), which contained protease inhibitor (1 mM phenylmethylsulfonyl fluoride; Aladdin, Shanghai, China). The resuspended solutions were sonicated (10 min; 300 W; 30-s sonication and 30-s pause) in an ultrasonic crushing apparatus (Scientz, Ningbo, China) placed in an ice bath. After centrifugation at 5439 *g* for 10 min, the supernatant of bacterial lysate was collected and solubility was detected by SDS-PAGE.

Following previously confirmed premium conditions for protein expression, the activated bacteria were incubated as 1% of the inoculum in an LB medium. The supernatant was collected after sonication and centrifugation of the bacterial cells. The supernatant containing the soluble recombinant protein was purified using Ni–NTA His Bind Resin (7sea Biotech, Shanghai, China). After protein binding with the column, the washing buffer (20 mM Tris, 250 mM NaCl, 20 mM Imidazole; Aladdin) and the elution buffer (20 mM Tris, 250 mM NaCl, 200/500 mM Imidazole) were used to elute the target recombinant proteins. The purified target proteins were identified by SDS-PAGE and western blot. In the western blot analysis, mouse Anti-His monoclonal antibody (Boster Biotech, Wuhan, China) was used as the primary antibody (1:2,000 dilution in Tris-buffered saline) and goat anti-mouse IgG labeled with horseradish peroxide (1:5,000 dilution in Tris-buffered saline) (Boster Biotech) was used as the secondary antibody.

### Measuring the effects of rHSC70 and rHSC70^TKD^ on blood coagulation

All animal experiments were approved and overseen by the Institutional Animal Care and Use Committee at Hunan Agricultural University (HUNAU; Hunan, China). Seven-week-old female Sprague Dawley (SD) rats were purchased from the Hunan SJA Laboratory Animal Co., Ltd. (Hunan, China). All rats who passed the quality inspection by the Hunan Anshenmei Pharmaceutical Research Institute Co., Ltd. (Hunan, China) were given sterile water and kept at 25 °C, 45% relative humidity for 7 days. Blood samples were collected from the orbital vein of six rats and mixed with 3.8% sodium citrate (citrate:blood: 1:9, v/v). Plasma was separated from the blood by centrifugation at 775.2 *g* for 10 min and stored at 4 °C within 2 h for further use to detect plasma anticoagulation [27]. To evaluate the dose–response anticlotting activities of rHSC70 and rHSC70^TKD^, harvested rHSC70 and rHSC70^TKD^ proteins were diluted with normal saline to concentrations of 0.5, 1, 2, 4 and 8 mM and stored at 4 °C. Bovine serum albumin (BSA; Aladdin) solutions were prepared at the same concentrations, and these solutions then mixed with rat plasma in equal volumes; these solutions served as the control groups. Different concentrations of recombinant proteins were mixed with rat plasma in equal volumes as the rHSC70 or rHSC70^TKD^ groups. Inhibitor VER155008 (100 μM; ApexBio Technology, Houston, TX, USA), which is an effective inhibitor of rHSC70 [28, 29], was added to different concentrations of recombinant protein solutions and mixed with an equal volume of rat plasma as the inhibitor group. All samples and reagents were preheated to 37 °C before determination.

A MC-1000 coagulometer (TECO Medical Instruments, Niederbayern, Germany) was used to measure the thrombin time (TT), fibrinogen (FIB) content, prothrombin time (PT) and activated partial thromboplastin time (APTT) of the samples according to the instructions of the commodity kit (Sun Biotech, Shanghai, China). Each group was measured eight times in parallel. In the PT test, a 100-μl sample was added to a cuvette at 37 °C for 2 min, then incubated with 200 μl PT reagents to record the time. In the TT test, a 200-μl sample was mixed with 200 μl TT reagents in a cuvette to record the time. In the APPT test, a 100-μl sample was mixed with 100 μl APTT reagents and incubated in a cuvette at 37 °C for 5 min, then added to 100 μl CaCl_2_ (25 mM) to record the time. In the FIB test, a 200-μl sample was mixed with 100 μl reagents to record the time. A standard curve was created first using reference plasma of known FIB content; then, the diluted samples (sample:normal saline = 1:9, v/v) were mixed with FIB reagents to record the time. FIB content was calculated based on the standard curve.

### Evaluation of rHSC70 and rHSC70^TKD^ immunogenicity

rHSC70, rHSC70^TKD^ and saline were emulsified with complete and incomplete Freund’s adjuvants (antigen:adjuvant = 1:1, v/v; Sigma Aldrich, Saint Louis, USA) using an aseptic device consisting of two syringes linked by a rubber infusion hose (length: 1 cm). Six female SD rats were placed in each group and injected with emulsified antigens of rHSC70 or rHSC70^TKD^ (50 µg recombinant protein was emulsified in an equal volume of adjuvant for each injection). Six separate female SD rats were injected with emulsified saline (the dosage of saline each time was the same as the volume of recombinant protein solution) and used as the negative control. For the first immunization, emulsified rHSC70, rHSC70^TKD^ and saline with complete Freund’s adjuvants were administered subcutaneously at multiple points on the backs of the rats. For the second and third immunizations, emulsified rHSC70, rHSC70^TKD^ and saline with incomplete Freund’s adjuvants were immunized at the same site on the rat backs. The interval between each immunization was 2 weeks. Blood was collected from the orbital vein 2 weeks after each immunization. The serum samples were released, centrifuged and stored at − 20 °C.

The serum antibody titer was detected using indirect enzyme-linked immunosorbent assay (ELISA). The antigen (rHSC70/rHSC70^TKD^) was diluted with ELISA coating solution (Bioss Biotech, Beijing, China) to a concentration of 40 µg/ml. Each well of the microplate (Boster Biotech) contained 100 μl antigen solution, which was immobilized by incubation at 37 °C for 2 h. The wells were washed four times with ELISA washing solution (Bioss Biotech), then incubated with 200 μl ELISA blocking solution (Bioss Biotech) at 37 °C for 1 h, following which the blocking solution was subsequently aspirated off the microplate. The rat serum from the rHSC70/rHSC70^TKD^ group was diluted in a gradient in phosphate buffered saline (PBS; Aladdin) (pH 7.4, 137 mM NaCl, 2.7 mM KCl, 10 mM KH_2_PO_4_, ddH_2_0 volume of 1 L), and the negative group serum was diluted at 1:1,000 in PBS, which was used as the primary antibody at 100 μl per well and incubated at 37 °C for 2 h; two wells containing 100 μl of PBS alone were set to assess the enzymatic reaction. The wells were washed four times with ELISA washing solution. Goat anti-mouse IgG labeled with horseradish peroxide (1:5,000 dilution in ELISA antibody dilution buffer) (Bioss Biotech) was used as the secondary antibody at 100 μl per well and incubated at 37 °C for 2 h. The wells were washed four times with ELISA washing solution. Then, 100 μl of 3,3',5,5'-tetramethyl benzidine (TMB) two-component substrate solution (for ELISA) (Bioss Biotech) was added to each well and incubated in the dark at 37 °C for 5–15 min, following which 50 μl ELISA stop solution (Bioss Biotech) was added to each well. The absorbance was measured at OD_450nm_ on a plate reader (BioTek, Vermont, USA). The criterion for positivity was the (rHSC70/rHSC70^TKD^) group serum at OD_450nm_/the negative group serum at OD_450nm_ ≥ 2.1; otherwise, it was determined to be negative [30–32]. The maximum dilution of the positive reaction was regarded as the titer of the serum.

The serum concentrations of interferon-gamma (IFN-γ) and interleukin-4 (IL-4) in all groups were measured using ELISA kits (Ruixinbio, Quanzhou, China) for rats according to the protocols provided by the manufacturers [33]. All analyses were performed using a plate reader and the results were expressed. The standard sample supported by the kits and its measurements at OD_450nm_ were used to obtain the standard curve line using the four-parameter logistic curve-fitting model. Cytokine concentrations were calculated based on the standard curve.

### Homology analysis

The amino acid sequence of HSC70 of *H. flava* was aligned with other species of HSPs. All the amino acid sequences used in this research were retrieved from the NCBI Protein Database (https://www.ncbi.nlm.nih.gov/protein/). The homology analysis was conducted using the Multiple Sequence Alignment by Clustal W (https://www.genome.jp/tools-bin/clustalw/), and the analysis results were visualized in a graphic format with ESPript 3.0 (https://espript.ibcp.fr/ESPript/ESPript/).

## Results

### Cloning of the HSC70 ORF of *H. flava *and site-directed mutation

**Fig. 1:**
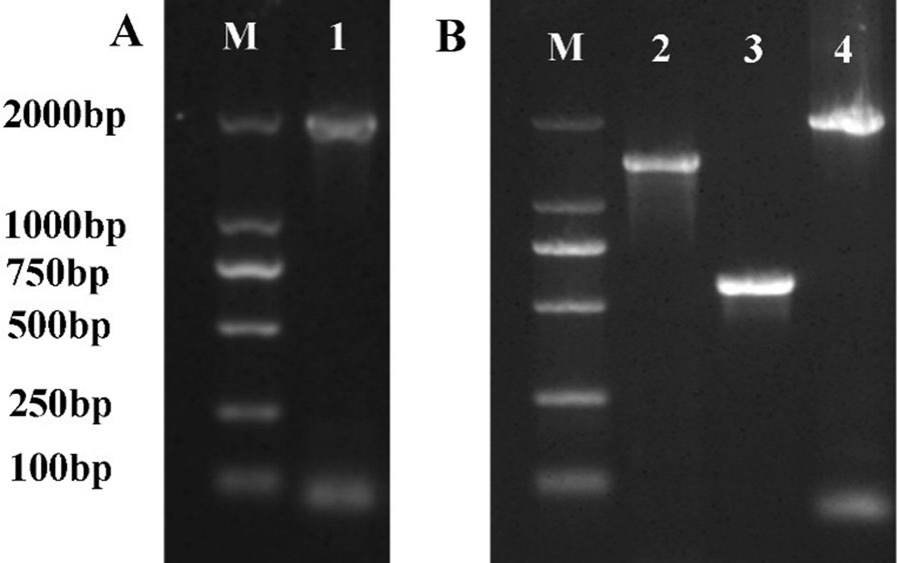
The open reading frame (ORF) of heat shock cognate 70 (HSC70) amplification products and of the TKD peptide mutation (HSC70^TKD^) amplification products. **A** Agarose gel electrophoresis of the ORF of HSC70 amplification products cloned from *Haemaphysalis flava*. **B** Agarose gel electrophoresis of HSC70^TKD^. Lanes: M, DNA marker; 1, products of the HSC70 gene ORF; 2, mutant gene fragments with T1 in front; 3, mutant gene fragments with T2 in the back; 4, HSC70^TKD^ amplification products

**Fig. 2:**
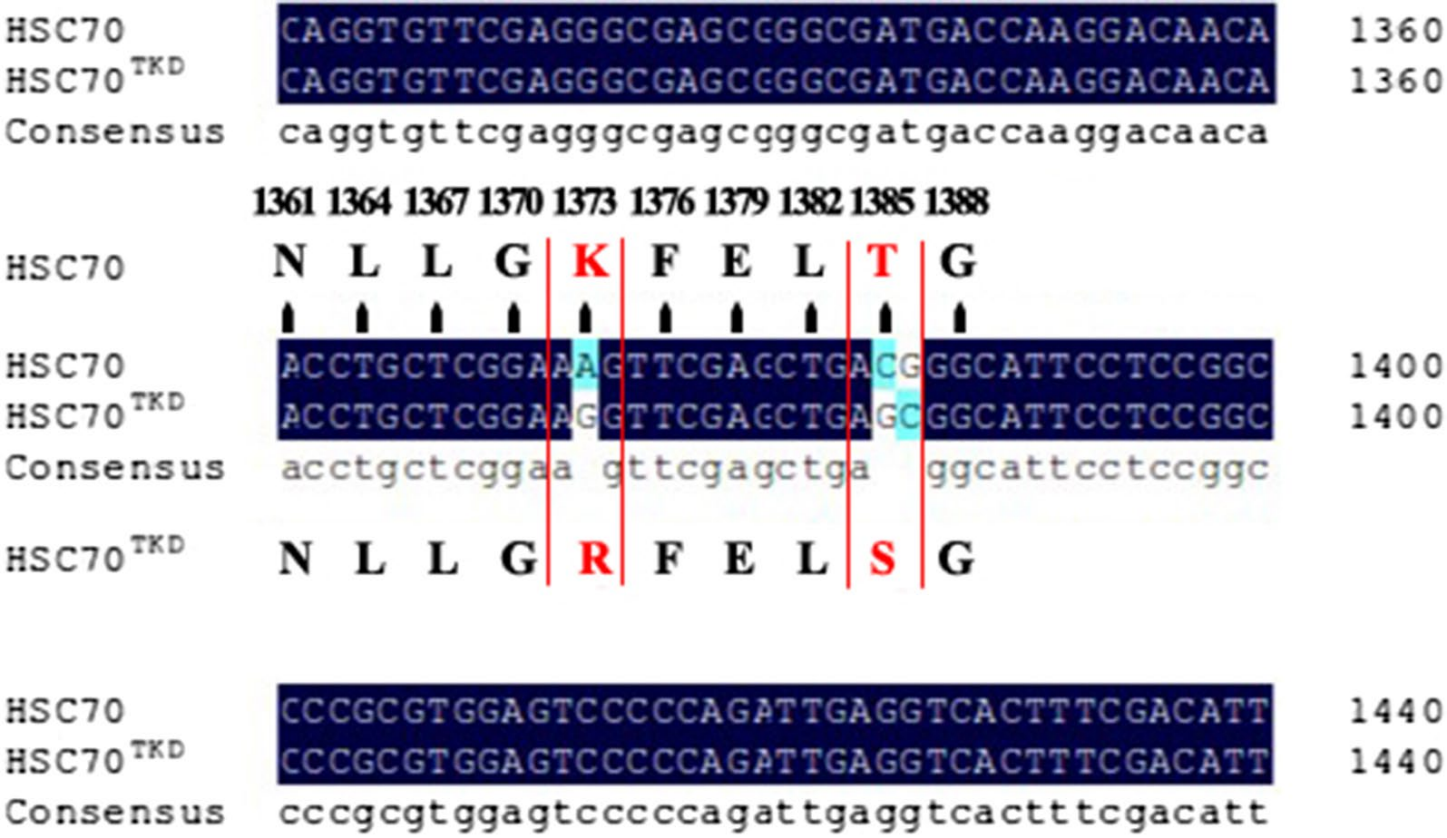
Multiple sequence alignment of the partial length of the HSC70 gene coding the TKD peptide between the PCR amplification products of HSC70 and HSC70^TKD^ (visualized with DNAMAN)

The length of the HSC70 ORF of *H. flava* was 1958 bp, which was consistent with our expectations (Fig. 1A). The lengths of the T1 and T2 sequences were 1397 and 583 bp, respectively (Fig. 1B). The HSC70^TKD^ ORF was successfully amplified using the overlap extension method (Fig. 1B). Comparison of the two sequences revealed that the 1373-position adenine (A) of the HSC70 ORF was mutated into guanine (G), the 1385-position cytosine (C) was mutated into G, and the 1386-position G was mutated into C. The gene encoding the TKD-like peptide (TKDNNLLGKFELTG) of HSC70 was artificially mutated into the TKD peptide (TKDNNLLGRFELSG) (Fig. 2).

### Expression and affinity purification of rHSC70 and rHSC70^TKD^

Restriction endonuclease digestion of the recombinant plasmids pET-28a-HSC70 or pET-28a-HSC70^TKD^ obtained a product of 2000 bp in size, which was consistent with the target fragment length (1958 bp), suggesting that the ORFs of HSC70 and HSC70^TKD^ were successfully inserted into pET-28a. Both pET-28a-HSC70 and pET-28a-HSC70^TKD^ were expressed in *E*. *coli* at 0.5 mM IPTG. Following SDS-PAGE we detected robust proteins at a molecular weight of 70 kDa in whole cell lysates. The expression of both plasmids reached their maximum level after 6 h. In terms of soluble expression, rHSC70 was found to be more highly expressed in the supernatant than in the precipitated fraction while rHSC70^TKD^ was expressed less in the supernatant than in the precipitated fraction.

**Fig. 3:**
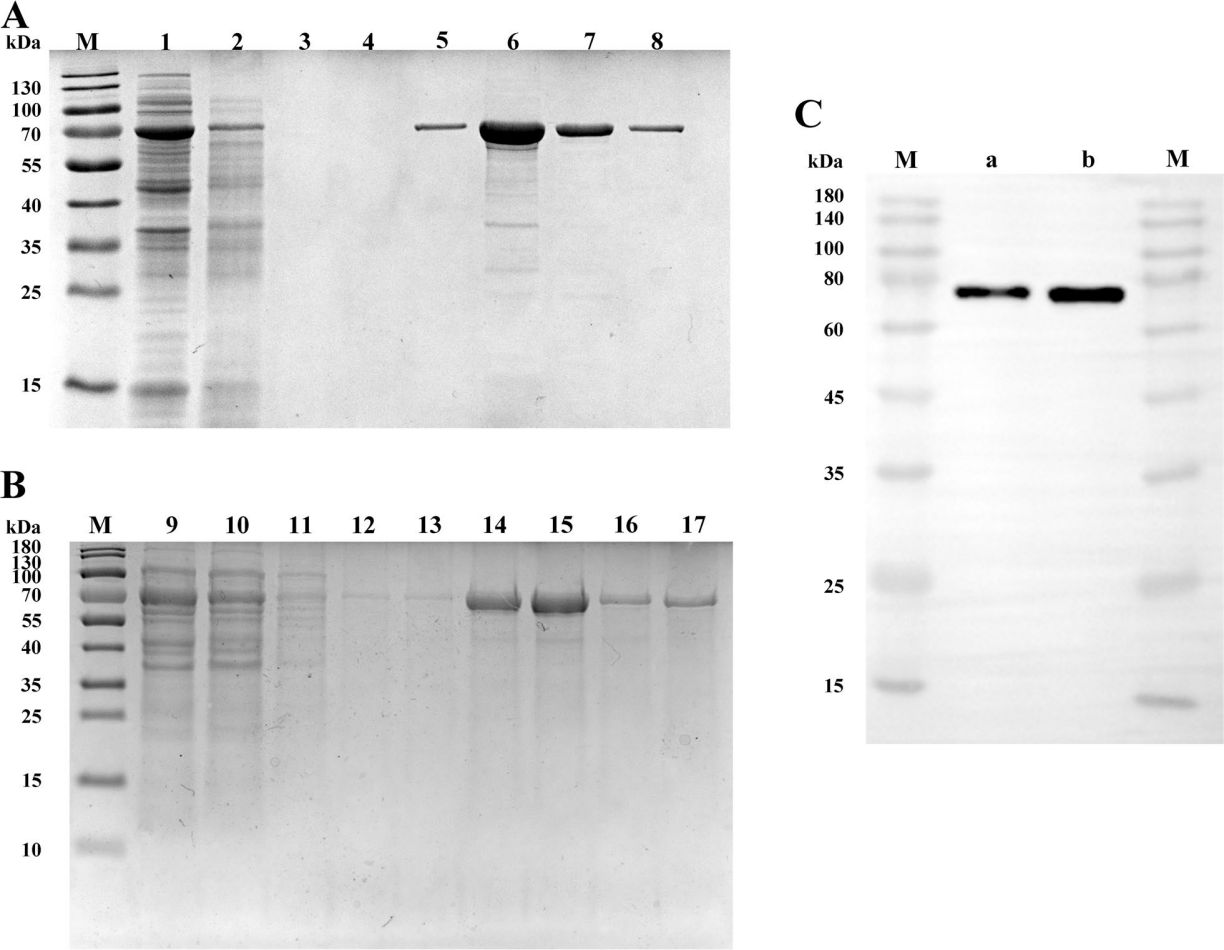
Affinity purification results of the recombinant proteins separated by passage through a nickel column. **A** Sodium dodecyl sulfate–polyacrylamide gel electrophoresis (SDS-PAGE) analysis of purified recombinant protein HSC70 (rHSC70). **B** SDS-PAGE analysis of purified recombinant protein HSC70^TKD^ (rHSC70^TKD^). **C** Identification of rHSC70 and rHSC70^TKD^ by western blot. Lanes: M, Protein marker; 1, 9, the supernatant of rHSC70 and rHSC70^TKD^ after bacterial destruction, respectively; 2, 10, rHSC70 and rHSC70^TKD^ passing through the column, respectively; 3, 4 and 11–13, rHSC70 and rHSC70^TKD^ 20 mmol/l eluent, respectively; 5–8 and 14–17, rHSC70 and rHSC70^TKD^ 200 mmol/l eluent, respectively; a. purified rHSC70; b. purified rHSC70^TKD^

After purification with the Ni–NTA His Binding Resin, the electrophoresis band of the eluate was thick and clear (Fig. 3, lanes 6 and 15), suggesting that most of the target proteins were eluted, the impurity proteins were low in number and the purities of the target proteins were high (Fig. 3A, B). The western blot results showed a single protein band at 70 kDa in the proteins extracted from the IPTG-induced culture medium (Fig. 3C). The elute contained target proteins that were further concentrated using a dialysis bag, yielding rHSC70 and rHSC70^TKD^.

### Effects of rHSC70 and rHSC70^TKD^ on plasma coagulation

**Fig. 4:**
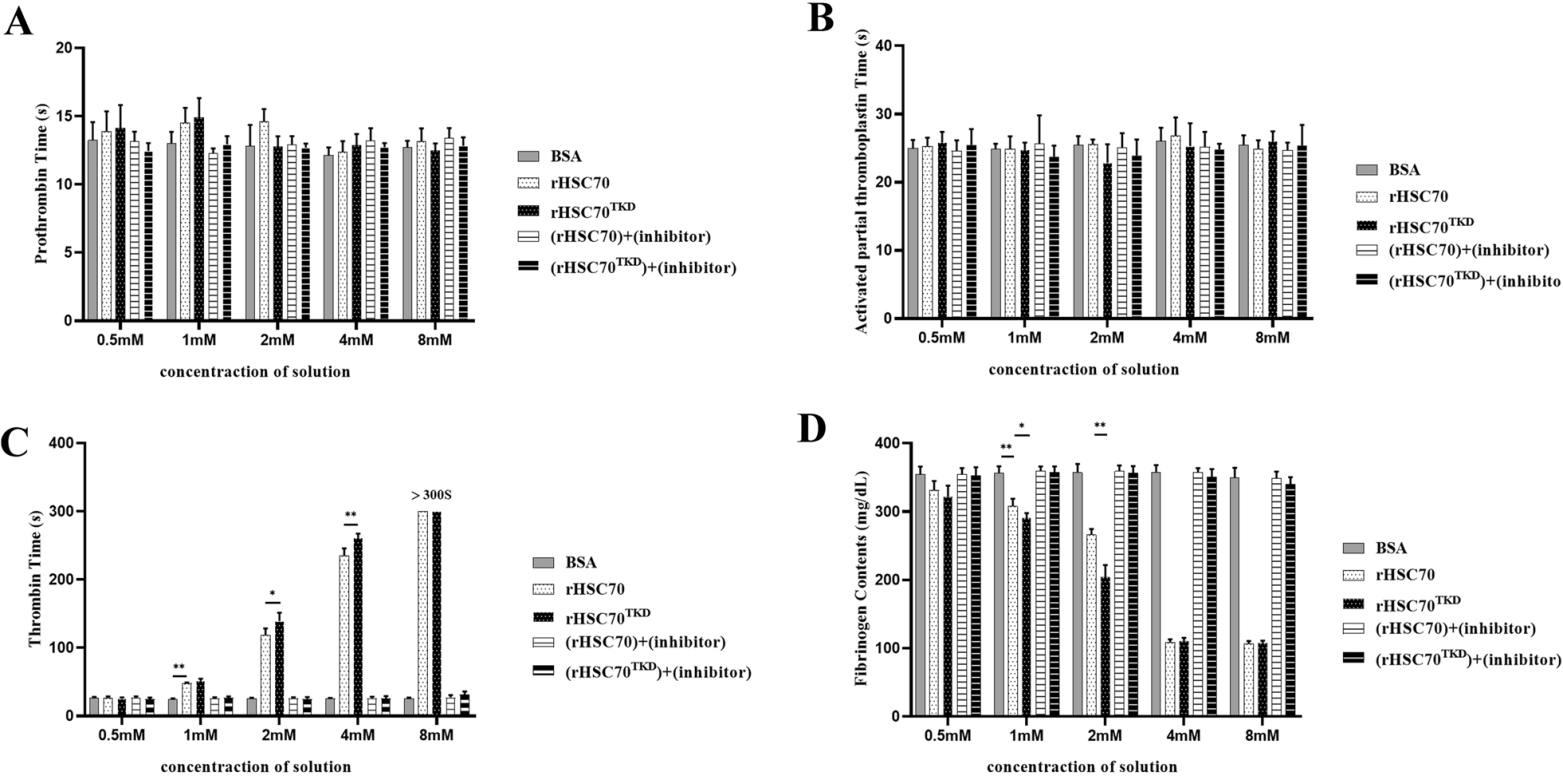
Effects of rHSC70 and rHSC70^TKD^ on the four coagulation parameters. **A** Effects of different concentrations of rHSC70 and rHSC70^TKD^ on the prothrombin time, **B** Effects of different concentrations of rHSC70 and rHSC70^TKD^ on the activated partial thromboplastin time, **C** Effects of different concentrations of rHSC70 and rHSC70^TKD^ on the thrombin time,** D** Effects of different concentrations of rHSC70 and rHSC70^TKD^ on the fibrinogen content. BSA solutions with concentrations comparable to rHSC70 were used as the negative controls. The inhibitor (VER155008) was mixed with recombinant proteins to a final concentration of 100 μM. > 300 indicates that the measured time exceeds the maximum range of the coagulometer by 300 s; Asterisks indicate significant differences between groups at *P < 0.05 and **P < 0.01. Eight replicates were used for each group of data in parallel. BSA, Bovine serum albumin

Neither rHSC70 nor rHSC70^TKD^ inhibited rat plasma coagulation in vitro through an effect on the PT or APTT (Fig. 4A, B). When the concentrations of rHSC70 and rHSC70^TKD^ were 1 mM, the TT test was significantly lengthened (*P* < 0.01), and the FIB content was significantly lowered (*P* < 0.01), when compared to the control group (Fig. 4C, D). With increasing concentrations of rHSC70 and rHSC70^TKD^, the TT was further prolonged and the FIB content was further reduced (Fig. 4C, D). rHSC70 and rHSC70^TKD^ no longer had the ability to inhibit blood coagulation at different concentrations due to the presence of inhibitors (Fig. 4C, D). These results showed that rHSC70 and rHSC70^TKD^ played anticoagulant roles by inhibiting the TT and reducing the FIB content.

The anticoagulant efficiencies of rHSC70 and rHSC70^TKD^ were different as the concentration increased. In the TT test, the anticoagulant efficiency of rHSC70^TKD^ was significantly higher than rHSC70 at 2 mM (*P* < 0.05) and 4 mM (*P* < 0.01) (Fig. 4C). In the FIB test, the anticoagulant efficiency of rHSC70^TKD^ was significantly higher than rHSC70 at 1 mM (*P* < 0.05) and 2 mM (*P* < 0.01) (Fig. 4D). These results showed that the ability of rHSC70^TKD^ to inhibit the TT and reduce the FIB content in vitro was better than that of rHSC70.

### Antibody titers of rHSC70 and rHSC70^TKD^

The results of the study on the serum antibody titers of rHSC70 and rHSC70^TKD^ are shown in Table 2. After the first immunization, the serum antibody titer of the rHSC70 group and rHSC70^TKD^ group reached 1:8,000 and 1:4,000, respectively, indicating that both rHSC70 and rHSC70^TKD^ possessed certain levels of immunogenicity. After the second immunization, the serum antibody titer of the rHSC70 group reached 1:32,000 and that of the rHSC70^TKD^ group reached 1:16,000. After the third immunization, the serum antibody titers of rHSC70 and rHSC70^TKD^ further increased, and both reached 1:124,000.

**Table 2:** Determination of serum titer by indirect competitive enzyme-linked immunosorbent assay

Sample		Various dilutions of positive sera								Negative	Blank	Titer
Group	Immunization times	1000	2000	4000	8000	16,000	32,000	64,000	124,000			
rHSC70	First	3.826	3.499	3.127	2.126	1.296	0.850	0.576	0.493	0.961	0.146	8000
	Second	3.608	3.472	3.221	2.802	1.932	1.664	1.431	1.375	0.785	0.152	32,000
	Third	3.798	3.723	3.768	3.723	3.492	2.974	2.570	1.946	0.677	0.083	124,000
rHSC70^TKD^	First	2.865	1.500	0.807	0.471	0.250	0.174	0.158	0.216	0.271	0.147	4000
	Second	2.993	2.272	2.301	0.894	0.657	0.349	0.228	0.195	0.265	0.133	16,000
	Third	3.244	3.188	3.056	2.205	0.963	0.713	0.656	0.567	0.289	0.131	124,000

rHSC70 Recombinant heat shock cognate 70, rHSC70^TKD^ Recombinant protein HSC70 (TKD peptide mutation)

### Cytokine concentrations

**Fig. 5:**
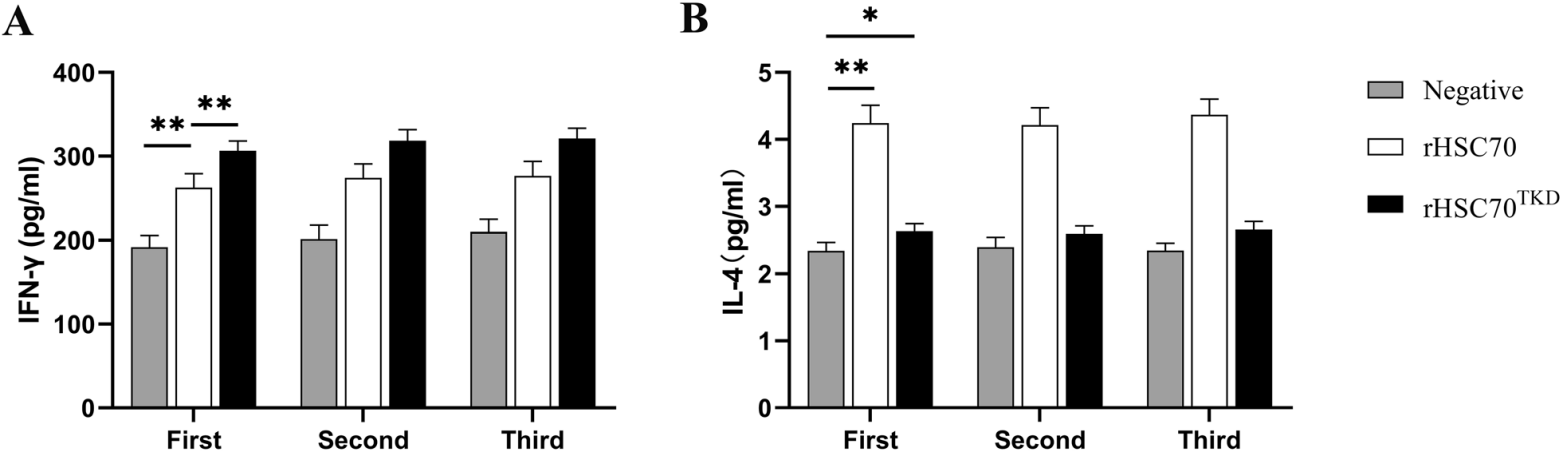
Effects of rHSC70 and rHSC70^TKD^ on the concentrations of rat serum cytokines.** A** IFN-γ in serum,** B** IL-4 in serum. First, Second, Third indicate immunization times. Bars represent mean ± standard error of the mean (*n* = 6). Asterisks indicate significant differences between groups at *P < 0.05 and **P < 0.01, respectively. IFN-γ, Interferon-gamma; IL, interleukin

The serum concentrations of IFN-γ in the rHSC70 and rHSC70^TKD^ groups were significantly higher than those of the negative group after each immunization (*P* < 0.01) (Fig. 5A). The serum concentration of IFN-γ in the rHSC70 immunized rats was significantly lower than that in rHSC70^TKD^ immunized rats (*P* < 0.01) (Fig. 5A). In comparison with the negative and rHSC70^TKD^ groups, the rHSC70 group had the highest serum concentration of IL-4 (*P* < 0.01) (Fig. 5B). The serum concentration of IL-4 in the rHSC70^TKD^ group was higher than that in the negative group (*P* < 0.05) (Fig. 5B).

## Discussion

The life-cycle of hard ticks includes the egg, larval, nymph and adult phases, and each active phase requires blood for growth and molting [34]. During blood-sucking and blood-meal digestion, it is essential that the tick is able to prevent blood coagulation. Especially during blood-meal digestion, the blood meal needs to be stored in the midgut in a flow state [29]. Subsequently, blood cells or hemoglobin are engulfed intracellularly in the digestive cells of ticks to provide nutrients for growth, development and reproduction [35], indicating that the basis of tick nutrition intake is to prevent blood coagulation and store the non-coagulated blood meal. Previous studies showed that the expression of HSC70 mRNA in ticks reached its highest level at the beginning of blood-feeding and remained at this high level until the semi-engorged status had been reached; in addition, the expression of HSC70 mRNA was significantly upregulated compared to that of unfed ticks [17, 36]. In one study on ticks, following silencing of HSP70 using the interference RNA method, the attachment and average engorgement rates of these ticks on the host decreased significantly and the ticks suffered a mortality rate as high as 97.44% [37]. These findings suggest that HSC70 plays an important anticoagulation role during tick blood-sucking and blood-meal digestion.

In the present study, we found that rHSC70 played an anticoagulant role in vitro through prolonging the TT and reducing the FIB content, which is consistent with the findings of a previous study [29]. Coagulation and anticoagulation are a dynamic physiological balance in which multiple factors of the body’s coagulation system participate in maintaining blood flow and preventing vascular thrombosis [38]. A previous study showed that HSP70 family proteins form phosphorylated complexes with other proteins during phosphorylation, and then the phosphorylated complexes bind with platelets to maintain a resting state for the platelets [39]. Inhibition of the function of the platelets of HSP70 affects the secretion of collagen-related peptides that activate the collagen receptor and glycoprotein VI, reduces platelet integrin-α_Iib_β_3_ activation [40] and reduces the combination of platelets and FIB. In this study, rHSC70 inhibited the TT and blocked the conversion of FIB solubility, suggesting that HSC70 has a certain level of anticoagulant activities. However, the exact anticoagulation mechanism remains to be determined.

**Fig. 6:**
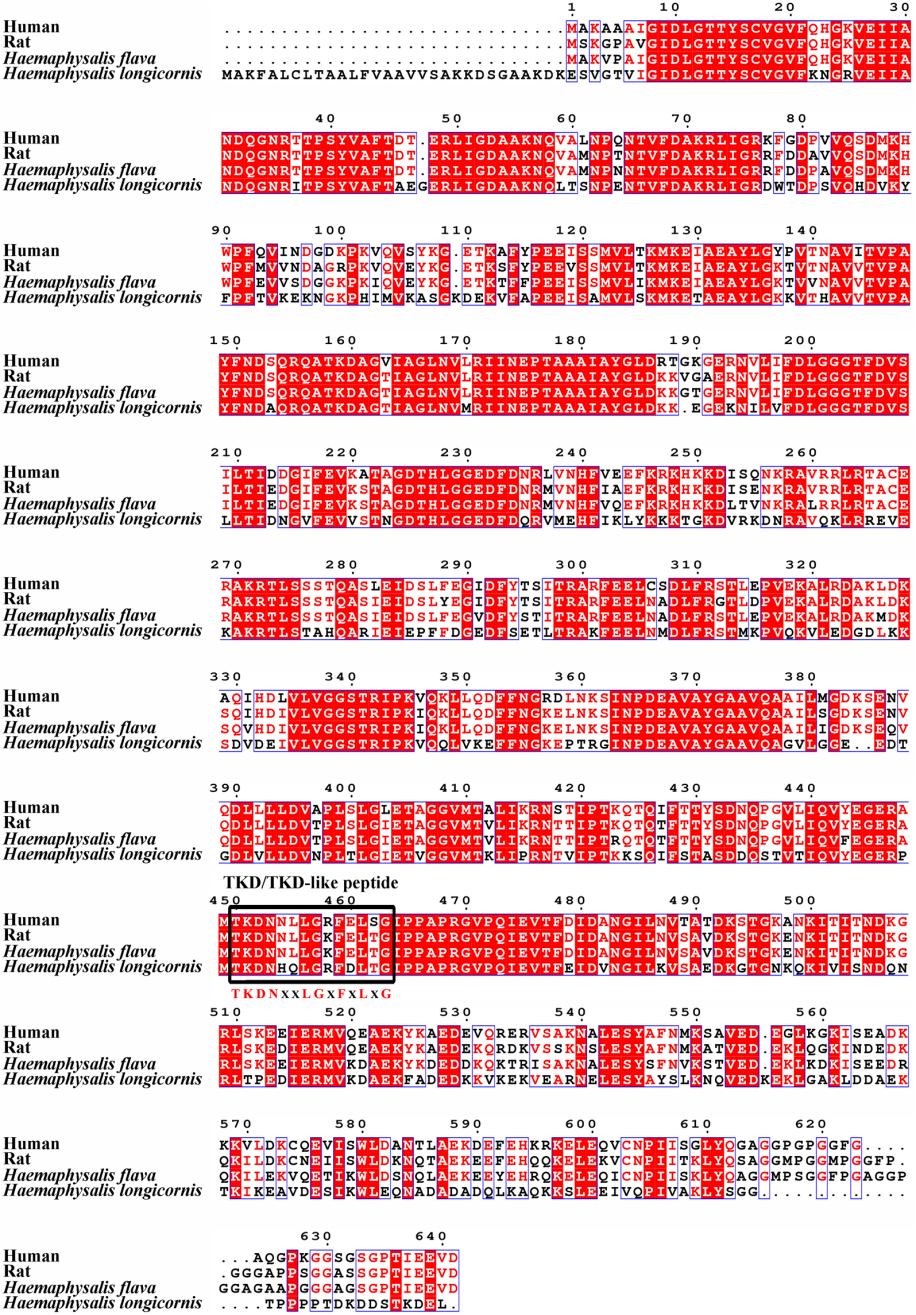
Comparison of amino acid sequences. Human indicates the sequence of heat shock 70 kDa protein 1A cloned from *Homo sapiens* (GenBank accession no. NP_005336). Rat indicates the sequence of heat shock cognate 71 kDa protein cloned from *Rattus norvegicus* (GenBank accession no. NP_077327). *Haemaphysalis flava* indicates the sequence of heat shock 70 kDa protein cloned from *Haemaphysalis flava* (GenBank accession no. AIS39468). *Haemaphysalis longicornis* indicates the sequence of heat shock 70 kDa protein 5 cloned from *Haemaphysalis longicornis* (GenBank accession no. ACA84007)

The immunogenicity of HSP70 cloned from *H*. *longicornis* was weak, as indicated by the low level of antibodies detected in rabbit sera [23]. However, previous studies found that some HSP70 family members cloned from other species, including *Schistosoma japonicum*, *Leishmania* and *Cryptosporidium andersoni*, possess better immunogenicity [18, 41, 42], especially the immunogenicity of recombinant C-terminal HSP70, which was found to be expressed the highest in different truncated forms of HSP70 cloned from *Leishmania major* [43]. We comparing the HSC70 gene sequences of *H*. *flava* obtained in this study and HSP70 sequences of *H. longicornis* published in the GenBank database in humans and rats (Fig. 6), and found that these C-terminal sequences were less conserved. Although no explicit explanation is available for C-terminal HSP70 function, we speculate that the C-terminal is related to the specific functions of HSP70 family members [14]. Compared with the results reported by Tian et al. [23], who cloned the HSP70 homologous protein, HSP70-5, and not HSC70 (HSP70-8), the sequences of HSP70-5 and HSC70 had only 64.6% homology. Our previous studies found that TKD-like peptides of HSP70 family members of ticks differed from human HSP70 TKD peptides, as they varied at individual amino acid positions [24]. The TKD peptide, a key immune peptide and domain of HSP70 at amino acids (aa) 450–463, proliferates the activity of NK cells and promotes the secretion of IFN-γ [25, 44], thereby stimulating the serum concentration of IFN-γ and IL-4 to counteract the tick self-protection mechanism [45, 46]. However, TKD-like peptides with individual base changes failed to stimulate immunity [25]; therefore, the TKD-like peptide of HSP70 in ticks does not have the same ability. To explore whether the immunogenicity of *H. flava* HSC70 could be enhanced by mutating its TKD-like peptides to TKD peptides, the TKD-like peptide of the HSC70 ORF was mutated into the TKD peptide, and the ORF of HSC70^TKD^ was amplified. To ensure that HSC70^TKD^ still had anticoagulant activities, the anticoagulant detection in vitro of rHSC70^TKD^ obtained by the prokaryotic expression system was carried out. Compared to rHSC70, rHSC70^TKD^ possessed anticoagulant activities and had a stronger ability for inhibiting the TT and reducing the FIB content in the plasma.

We followed a program for immunizing SD rats with rHSC70 and rHSC70^TKD^ emulsified in complete and incomplete Freund’s adjuvants [47–49], with the aim to confirm the immunogenicity of rHSC70 and rHSC70^TKD^. The IFN-γ and IL-4 cytokines were core indicators to evaluate whether T helper (Th) cells had developed into Th1 or Th2 [50]. Activated CD4^+^ T cells, activated CD8^+^ T cells and activated NK cells can also produce IFN-γ [51, 52]. Previous studies showed that HSP70 can strengthen the expression of MHC II in antigen-presenting cells, such as macrophages and dendritic cells, which could encourage CD4^+^ T cells to produce IFN-γ for the immune response [22, 53]. In this study, we detected a higher serum concentration of IFN-γ in immunized rHSC70 and rHSC70^TKD^ rats compared with the negative control rats. This result is consistent with the results reported in earlier studies [22, 53]. However, the serum concentration of IFN-γ in the rHSC70^TKD^ immunized rats were significantly higher that in the rHSC70 immunized rats. It is known that the production of IFN-γ is associated with the co-secretion of various immune cells; therefore, the TKD peptides may be essential for an increased production of IFN-γ [25, 44, 54]. The serum concentration of IL-4 was higher in the rHSC70^TKD^ immunized rats than in the negative rats, with the rHSC70 immunized rats having the highest IL-4 concentration. It is possible that some Th cells could be differentiated into Th2 cells, and a lower number of Th2 cells produce a low serum concentration of IL-4 [22, 55, 56]. After the first and second immunizations, both the rHSC70 and rHSC70^TKD^ groups showed strong humoral immune responses, and the serum antibody titers in the rHSC70^TKD^ group were lower than those in the rHSC70 group. The antibody titers and IL-4 concentrations of the rHSC70 and rHSC70^TKD^ groups were positively correlated at the first and second immunizations, possibly because IL-4 can promote B cell differentiation and antibody production [57]. Strengthened by the third immunization, the serum antibody titers of the two groups were further enhanced and reached 1:124,000. These results showed that both rHSC70- and rHSC70^TKD^-stimulated animals produce higher antibody levels and higher levels of cellular immunity. Our results indicated that the immunogenicity of *H*. *flava* HSC70 and HSC70^TKD^ was strong.

## Conclusion

This study proves that rHSC70 and rHSC70^TKD^ can significantly prolong the TT and reduce the FIB content in plasma, and that rHSC70^TKD^ has stronger anticoagulant activities than rHSC70. Both rHSC70 and rHSC70^TKD^ can stimulate rats to produce high antibody levels and higher levels of cellular immunity. Both of them have good immunogenicity.

## Data Availability

Not applicable.

## Abbreviations

APTT: Activated partial thromboplastin time
ELISA: Enzyme-linked immunosorbent assay
FIB: Fibrinogen
HSC70: Heat shock cognate 70
HSP: Heat shock protein
HSP70: Heat shock protein 70
IFN-γ: Interferon-gamma
IL-4: Interleukin-4
IPTG: Isopropyl β-d-1-thiogalactopyranoside
MHC: Major histocompatibility complex
OD: Optical density
ORF: Open reading frame
PT: Prothrombin time
rHSC70: Recombinant protein HSC70
rHSC70^TKD^: Recombinant protein HSC70^TKD^
SDS-PAGE: Sodium dodecyl sulfate–polyacrylamide gel electrophoresis
TT: Thrombin time
